# Rethinking gestational diabetes: hyperglycemia or a cluster of cardiometabolic abnormalities manifested during pregnancy?

**DOI:** 10.20945/2359-3997000000519

**Published:** 2022-09-08

**Authors:** Maria Inês Schmidt

**Affiliations:** 1 Universidade Federal do Rio Grande do Sul Programa de Pós-Graduação em Epidemiologia Porto Alegre RS Brasil Programa de Pós-Graduação em Epidemiologia, Universidade Federal do Rio Grande do Sul, Porto Alegre, RS, Brasil; 2 Hospital de Clínicas de Porto Alegre Porto Alegre RS Brasil Hospital de Clínicas de Porto Alegre, Porto Alegre, RS, Brasil

Until the last century, diabetes and pregnancy did not materialize as clinical issues because women with type 1 diabetes almost inevitably died before getting pregnant, and women with type 2 diabetes during reproductive ages barely existed. The first report of diabetes in pregnancy, published in 1823, described a case of extreme fetal macrosomia, possibly related to diabetes. The following century accumulated more experience, and the belief that diabetes could, in some cases, be a “symptom” of pregnancy led to the concept of gestational diabetes (GDM) (1).

Decades of research after the 1950s have shown considerable implications of GDM in and beyond pregnancy. The iconic studies establishing diagnostic criteria for GDM during pregnancy used the incidence of future type 2 diabetes for validation (2). Based on this definition, rates of GDM during pregnancy and rates of glucose intolerance in non-pregnant states were remarkably similar, supporting the notion that GDM represents the discovery of a preexisting glucose intolerance (3). Currently, GDM is diagnosed based on pregnancy adverse outcomes, including large for gestational age, macrosomia, hypertensive disorders, cesarian section, and shoulder dystocia (4,5). Using these new criteria, GDM has become the most frequent clinical condition detected during prenatal care.

Of note, the epidemics of obesity and type 2 diabetes initiating over the last few decades have changed the nature of GDM. In addition to hyperglycemia, obesity and possibly also central obesity are likely strong players in the causation of adverse outcomes. With regard to central obesity, we reported in the 1990s that pregnant Brazilian women with high waist circumference and waist-to-hip ratio had higher glycemic levels at the time of GDM screening, particularly those whose uterine height had not reached the waist. This finding has been confirmed by others and recently summarized in a systematic review (6). Yet, the practicalities of routine waist circumference measurement in pregnant women have limited its clinical application.

In this issue of the AE&M, Carvalho and cols. used neck circumference (NC) to evaluate the relationship of fat distribution with GDM (7). They found a 25% increased risk of GDM related to an elevated NC. The cut-off of >34.5 cm identified 70% of those with GDM (sensitivity) and 49% of those without GDM (1-specificity). Women above this cut-off *vs*. the remaining ones also had higher frequencies of hypertension. Similar results were seen in a systematic review of various studies (6), and reported in additional ones (8–11), with optimal cut-offs ranging from 34 to 38 cm, and areas under the curve from 0.58 to 0.67.

The implications of these findings are noteworthy. GDM, like impaired glucose tolerance and metabolic syndrome, has been increasingly recognized as a risk factor for both type 2 diabetes and cardiovascular disease (CVD) in women (12), which is consonant with GDM representing the discovery, during pregnancy, of a glucose intolerance state present before pregnancy (3). As such, the diagnosis of GDM can be seen as an opportunity to detect high-risk women and initiate diabetes and CVD prevention. We are now finalizing a multicenter randomized clinical trial of lifestyle interventions after pregnancy to evaluate their effectiveness in diabetes prevention among women with recent GDM (13). Although identifying women at high risk was achieved, our interventions gained limited adherence, raising the need to focus on the subgroup at the highest risk.

Prediction scores to this end will need to be developed, considering in addition to glucose levels and use of antidiabetic medication in pregnancy, also other parameters of the complex metabolic array of factors related to GDM, including fat distribution. Waist circumference/neck circumference are good candidates for this end. However, additional factors must also be considered. For example, fatty liver indices, just beginning to be evaluated in pregnant women (14), may also interact with this complex metabolic path linking hyperglycemia, insulin resistance, obesity, central obesity, dyslipidemia, and more (15). Thus, GDM may also constitute a cluster of cardiometabolic factors, a metabolic syndrome manifested in pregnancy. Given the high risk of future diseases conferred by GDM, not only type 2 diabetes but also cardiovascular diseases, chronic kidney diseases, and other chronic conditions, such a vision of clustered metabolic abnormalities in pregnancy opens a window of opportunities for prevention we cannot afford to miss.
